# The Reasons for Doing Physical Exercise Mediate the Effect of Self-Esteem on Uncontrolled Eating Amongst Nursing Personnel

**DOI:** 10.3390/nu11020302

**Published:** 2019-01-31

**Authors:** María del Carmen Pérez-Fuentes, María del Mar Molero Jurado, María del Mar Simón Márquez, José Jesús Gázquez Linares

**Affiliations:** 1 Departament of Psychology, University of Almería, 04120 Almería, Spain; mmj130@ual.es (M.d.M.M.J.); msm112@ual.es (M.d.M.S.M.); jlinares@ual.es (J.J.G.L.); 2 Departament of Psychology, Universidad Autónoma de Chile, 4780000 Santiago, Chile

**Keywords:** eating, exercise, self-esteem, nursing

## Abstract

Background: Since the beginning of the 20th century, the importance of creating healthy work environments and promoting the health of workers in the healthcare sector to create Healthy and Resilient Organizations has been emphasized. In this context, self-esteem is an essential construct which influences health and healthy life styles, and, therefore, the general wellbeing of nurses. The objective of this study was to analyze the mediating role of reasons for exercising in the effect that self-esteem has on uncontrolled eating by nursing professionals. Methods: The sample was made up of 1094 nurses who were administered the Rosenberg General Self-Esteem Scale, the Goal Content for Exercise Questionnaire, and the Three-Factor Eating Questionnaire-R18. Results: Bivariate correlation analysis and multiple mediation analysis showed that self-esteem has direct and indirect effects on uncontrolled eating. Moreover, self-esteem determines whether one does physical exercise to improve one’s image, recognition, or social affiliation—although the effects on uncontrolled eating were only significant in the case of image. Conclusions: The results have important practical implications in the framework of Positive Occupational Health Psychology (POHP) as they emphasize self-esteem, physical exercise and eating as essential aspects of the health and wellbeing of employees in the healthcare sector, highlighting the importance of creating organizations committed to promoting the psychosocial health of their workers.

## 1. Introduction

From the beginning of the 20th century, with the rise of disciplines such as Occupational Health Psychology (OHP) and Positive Organizational Psychology (POP), the importance of creating healthy work environments and promoting organizations committed to the development and advancement of the psychosocial health of their workers has been emphasized (National Institute of Occupational Safety and Health, NIOSH) [1,2].

In this context, the Healthy and Resilient Organizations (HERO) model—a theoretical framework of risk evaluation directed at intervention [3]—is one of the most outstanding contributions of the Spanish WANT research team in Positive Occupational Health Psychology (POHP). HERO refers to “Those organizations which make systematic, planned, and proactive efforts to improve the health of their employees and the results of the organization” through healthy organizational practices [2]. From this perspective, a Healthy and Resilient Organization is characterized by developing practices for adequately managing work, promoting positive organizational results, and being able to depend on a healthy workforce [4]. Along this line, the model assumes that a healthy organization is not limited to understanding health in the workplace, but one that also takes into consideration the daily habits of workers’ lives [5]. 

At the present time, the study of self-esteem is one of the research areas that has awakened the most academic and professional interest due to its close relationship with physical and psychological wellbeing [6], social adjustment [7], and quality of life [8]. High levels of self-esteem are also related to optimum academic performance [9] and higher job performance and commitment [10,11]. It is an important protection factor against burnout in healthcare professionals [12]. On the contrary, low levels of self-esteem have been associated with depressive symptoms [13], anxiety [14], suicidal ideation [15], eating disorders [16], and violent behavior [17]. In brief, our overall self-assessment determines the way we are, the way we perceive the world, and the way we relate with others, and therefore influences our success in important facets of life [18,19]. According to the World Health Organization [20], nurses represent the largest group of healthcare professionals, but just because they know the importance of activities and behaviors that promote health does not mean that they have healthier living habits [21]. According to the HERO model, in the healthcare context, a healthy healthcare organization would be one that creates positive work climates contributing to greater productivity and makes an effort to maintain and improve the physical and psychological health of its employees. Promoting a healthy life style among employees is important because of its positive effect on their wellbeing as well as its consequences for the organization, i.e., the better the physical and mental health of the healthcare professionals, the higher the quality of the treatment offered to patients and their families will be [1,5]. Therefore, in recent years, there has been greater interest in studying eating and physical activity styles to prevent health-related problems (e.g., deficient clinical care, job absenteeism) [6,22].

It has been consistently demonstrated that physical exercise has considerable physical, psychological, and social benefits [23,24,25]. However, the objectives that people pursue when they do some type of activity have different cognitive, affective, and/or behavioral consequences. These different goals act as regulatory processes on their behavior which are essential to explain their starting and adhering to such activity [26]. In this sense, the Self-Determination Theory [27] provides a theoretical framework for studying the effects of the diverse motivations (intrinsic vs extrinsic) for becoming involved in a given physical activity. Intrinsic goals are directed at the search for affiliation, improved health, or personal growth [28]. These motivations are related to satisfying basic psychological needs (autonomy, competence and relatedness) [27] and function as a protection factor against anxiety and depression, contribute to greater subjective wellbeing and body satisfaction, and are associated with autonomous regulation of behavior [29]. On the contrary, extrinsic goals are directed rather at the desire to improve physical appearance or be popular and socially recognized. Unlike intrinsic goals, these respond to feelings of guilt, shame, and external pressure from the environment more than personal benefit itself, leading to interpersonal comparisons, which are a source of stress [28]. Therefore, extrinsic goals are not essential to wellbeing nor to personal development, since they are subject to social approval [29,30]. We might suggest that the level of self-esteem is a psychological factor of influence to goals attempted to be reached through physical exercise [31]. Thus, individuals with a positive view of themselves will seek stronger psychosocial development through activity and less satisfaction of external demands (e.g., sociocultural) [32]. Authors such as McAuley et al. [33] and Zamani Sani et al. [34] support the positive association between self-esteem and the practice of physical exercise. Specifically, Zamani Sani et al. [34] find a direct and indirect association of physical activity with self-esteem, although the authors themselves recognize the possibility of an inverse causality, i.e., that the level of self-esteem could have a certain degree of influence on the practice of physical exercise. In addition to the possible bidirectionality proposed by the scientific literature, the role of self-esteem as a mediating factor has also been analyzed in the link between physical activity and quality of life in young adults [35].

In another vein, regarding eating style, Booth et al. [36] have shown that the psychological mechanisms underlying unhealthy eating behavior must be known to develop prevention programs and promote health. Uncontrolled eating consists of the excessive consumption of food in response to external signals from the environment, characterized by a subjective increase in appetite and absence of self-control of eating behavior [37,38]. It negatively affects wellbeing and has been related with such health problems as obesity [39,40]. In this line, it has been demonstrated that ineffective emotional regulation strategies have a key role in starting and maintaining such eating behavior [41]. Moreover, people with an uncontrolled eating style show a tendency toward rumination and try to suppress undesirable moods [42]. Thus, uncontrolled eating is a mechanism by which the individual achieves a certain short-term emotional relief. This phenomenon may be interpreted more as the result of the individual’s effort to deviate attention from adverse emotions more than the satisfaction of eating in itself [42,43]. It has been specifically emphasized that negative emotions (e.g., sadness, anxiety, depressive symptoms), due to low self-esteem or other stressful factors are an antecedent of uncontrolled eating [43]. It has also been found that dissatisfaction with body image is linked to unhealthy eating habits and even predicts the development of eating disorders [44,45]. On the other hand, it is possible that individuals' reasons for exercise may make them more or less susceptible to uncontrolled eating (ingestion of unhealthy foods, for example) when faced with a stressor after exercise [46].

Because of the important implications that self-esteem, motivations for physical exercise, and eating behavior have on general wellbeing, our objective is to analyze the mediating role of the reasons for doing exercise in the effect that self-esteem has on uncontrolled eating in nursing professionals. We start from the following general hypothesis: the level of self-esteem maintains a direct relationship with uncontrolled eating, whereas the reasons for doing physical exercise play a mediating role of that link. We discuss aspects such as the differentiation of the reasons for physical exercise as well as the position of self-esteem within the proposed model.

## 2. Materials and Methods

### 2.1. Participants

The original sample was 1125 nurses in Andalusia (Spain). After discarding all incomplete questionnaires or ones with random answers (*n* = 31), the final study sample was 1094 nurses aged 22–57, with a mean age of 32.30 years (*SD* = 6.70). Of the total sample, 14.9% (*n* = 163) were men and 85.1% (*n* = 931) were women, with mean ages of 32.47 years (*SD* = 6.45) and 32.27 years (*SD* = 6.74), respectively.

### 2.2. Instruments

*Rosenberg Self-Esteem Scale* [47]. Developed for the evaluation of self-esteem in adolescents, it is made up of 10 items with contents focused on feelings of self-respect and self-acceptance. Answers are given on a four-point Likert-type response scale (from 1 = Strongly agree to 4 = Strongly disagree). Other studies have shown its adequate psychometric characteristics [48]. In this study, internal consistency was α = .82.

*Goal Content for Exercise Questionnaire* (GCEQ) [28]. This scale is made up of 20 items grouped in five factors: Health management (e.g., “To improve my overall health”), Image (e.g., “To improve my appearance”), Social recognition (e.g., (“To be socially respected by others”), Social affiliation (e.g., “To develop close friendships”), and Skill development (“To learn and exercise new techniques”). Answers are rated on a seven-point Likert-type scale (from 1 = not at all important to 7 = extremely important), where the subjects must rate to what extent the goals presented in the items are important to them while exercising. Internal consistency of the factors on the scale was calculated with the Cronbach’s alpha, finding .88 for Affiliation, .81 for Image, .87 for Health Management, .90 for Social recognition and .87 for Skill development. In previous studies, with a sample of adult women, the authors found that the alpha for factors varied from .72 to .85 [30].

*Three-Factor Eating Questionnaire-R18*. This is a brief version of the original 51-item TFEQ [49], translated and adapted to Spanish (TFEQ-SP) by Jáuregui-Lobera et al. [50]. In this study, the adaptation to a nursing population by Pérez-Fuentes et al. [51] was used. The question consists of 18 items rated on a four-point response scale (definitely true: 1, mostly true: 2, mostly false: 3, and definitely false: 4). It evaluates three dimensions of eating behavior: (a) Uncontrolled eating (the tendency to eat more than usual due to loss of control on eating with a subjective feeling of hunger); (b) Emotional eating (the inability to resist emotional signals or eating in response to negative emotions); and, (c) Cognitive restraint (the conscious restriction of eating directed at controlling body weight and/or promoting weight loss). The TFEQ-R18 has adequate reliability coefficients for the three subscales (varying from .75 to .87) [50] which are also adequate in a nursing population (.85 to .90) [51]. In this study, reliability indices were .89 on Uncontrolled eating, .84 on Emotional eating and .74 on Cognitive restraint. 

### 2.3. Procedure

Before data were collected, participants were guaranteed compliance with the standards of information, confidentiality, and ethics in data processing. The study was positively evaluated by the University of Almería Bioethics Committee. The questionnaires were implemented on a Web platform which enabled them to be filled out by participants online. To control random or incongruent answers, a series of control questions were included to detect them, and any such cases were discarded from the study sample. 

### 2.4. Data Analysis

First, bivariate correlation tests were done to check the relationships between the variables to be included in the causal analysis. The descriptive statistics for these variables were also found. 

The macro by [52] for Statistical Package for the Social Sciences (SPSS) was used to estimate the mediation model for, in this case, multiple mediation effects [53]. This resource enabled different regression models to be computed to find information on indirect effects, thus avoiding the limitations of the classical test [54]. For this, bootstrapping (10,000 bootstrap samples) was used, making it possible to estimate at 95% confidence intervals and determine the multiple mediating effect of the mediator variables. In this study, a multiple mediation analysis was carried out with three mediator variables forming a causal chain. 

## 3. Results

### 3.1. Descriptive and Correlation Analyses

Table 1 shows the descriptive and correlation statistics for variables: Global self-esteem, reasons for doing physical exercise, and uncontrolled eating. The data in table 1 confirm the existence of a negative correlation (*r* =-.20, *p* <.001) between the predictor variable (self-esteem) and uncontrolled eating as the dependent variable. Of the variables considered potential mediators (e.g., reasons for doing physical exercise), those with positive correlations with the dependent variable are image (*r* =.18, *p* <.001), social recognition (*r* =.13, *p* <.001), and social affiliation (*r* =.05, *p* <.05). These are the variables which were therefore included in the model as mediators.

### 3.2. Multiple Mediation Model for Estimating Self-Esteem as a Predictor and Mediation Effect Paths of Reasons for Physical Exercise on Uncontrolled Eating

The mediation analysis was carried out based on the following mediation hypothesis: Self-esteem level has repercussions on the performance of physical exercise motivated by image, and this, in turn, has a facilitating effect on uncontrolled eating. Social recognition and affiliation are reasons for exercising related to self-esteem, but they do not have any indirect effects on uncontrolled eating by this path. 

To compute Mediation 1, self-esteem was taken as the independent variable, and image, social recognition, and social affiliation were taken as the mediator variables (*M_1_*: IM, *M_2_*: S-RE, and *M_3_*: S-AF). 

**Figure 1:**
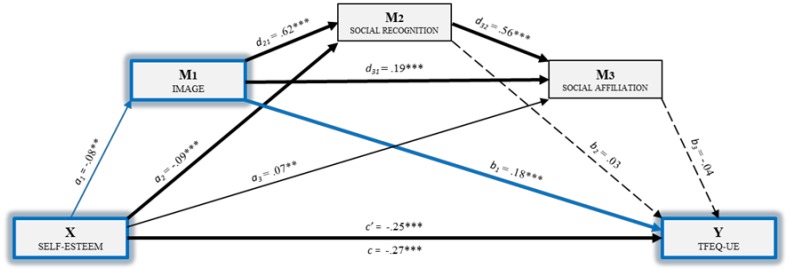
Multiple mediation model of reasons for doing physical exercise in the relationship between self-esteem and uncontrolled eating. Note: X = Independent variable: Self-esteem; M_1_= Mediator variable 1: Image; M_2_= Mediator variable 2: Social recognition; M_3_= Mediator variable 3: Social affiliation; Y= Dependent variable: Uncontrolled eating. ** *p* < .01; *** *p* < .001.

Figure 1 shows the multiple mediation model of uncontrolled eating, including the direct, indirect and total effects.

First, a statistically significant effect [B= -.08, *p* < .01] of self-esteem (X) on image (M_1_) is observed. The second regression analysis took Mediator 2 as the result variable and included self-esteem (X) and image (M1) in the equation. There was a significant effect of image [B = .62, *p* < .001] and of self-esteem [*B* = -.09, *p* < .001] on social recognition (M_2_). With the third regression analysis, which takes the result of social affiliation (M_3_) as the variable, the effect of the independent variable and of the other two mediators was estimated. Significant effects were observed in all cases: Self-esteem [*B* = .07, *p* < .01], image [*B* = .19, *p* < .001], and social recognition [*B* = .56, *p* < .001]. Moreover, of the three mediators, only image [*B* = .18, *p* < .001] showed significant effects on uncontrolled eating (*Y*). The direct effect of self-esteem on uncontrolled eating (*Y*) was significant [*B* = −.25, *p* < .001], and the total effect of model *B* = -.27, *p* < .001.

Finally, the analysis of the indirect effects was carried out with bootstrapping, which found data supporting a significant level for Path 1 [ind_1_: X→M_1_→Y; B= −.01, SE=.007, 95% CI (−.032, −.004)] (See Table 2).

## 4. Discussion

Self-esteem is one of the most studied personal constructs in the scope of an organization, because of its close relationship to wellbeing and quality of life. Empirical research has been particularly interested in its study in nursing professionals—a group especially vulnerable to burnout due to the characteristics of the work setting where they perform their functions [6,8,12]. Self-esteem is also involved in adopting healthy living habits, such as an adequate eating style and doing physical exercise. This prevents the appearance of health-related problems that can negatively affect the service quality offered and increase worker absenteeism [5,22]. Our results have shown that self-esteem has a direct effect on uncontrolled eating, which suggests that the negative effect characteristic of individuals with low levels of self-esteem facilitates an eating style which functions as an ineffective emotional regulation mechanism [42,43].

Furthermore, the data show that the level of self-esteem determines the goals which individuals attempt to reach through physical exercise; that is, individuals with a positive view of themselves tend to seek greater psychosocial development through social affiliation. According to the Self-Determination Theory (SDT) [27], social affiliation is an intrinsic goal associated with satisfying basic psychological needs. It positively affects the subjective wellbeing of individuals and acts as a protection factor against anxiety and depression [29]. Therefore, nursing professionals intrinsically motivated to do physical exercise show more adherence to this activity and have healthier life styles. It was also found that nursing professionals with high levels of self-esteem are usually less motivated by extrinsic goals directed at physical appearance and achieving more social recognition by doing physical exercise. These results support the idea that a positive overall self-evaluation leads to more personal accomplishment and less exercising in response to external demands [29]. Previous studies have also found significant differences in motivation by sex, showing that men give higher priority to social aspects related to physical exercise, while women show more concern for their image, which suggests that social pressure exerted by the media through its continual diffusion of stereotyped beauty may cause women to feel the need to be valued by society [30,44].

Results of the mediation models confirmed our hypotheses. Firstly, the level of self-esteem influenced doing exercise to improve image, and this positively affected uncontrolled eating. Though low levels of self-esteem led to exercising motivated by care of personal image, this type of motivation—far from improving body satisfaction and subjective wellbeing of individuals—is a source of stress and anxiety, since it refers to goals that are subject to social acceptance or approval [29]. As a consequence, individuals respond to that emotional distress by adopting an uncontrolled eating style to suppress the adverse emotions experienced. However, uncontrolled eating is an inadequate emotional regulation mechanism; that is, although individuals may feel a certain short-term emotional relief, in the long term it leads to increasing negative thoughts and health-related problems such as obesity [39,42,43]. Secondly, even though self-esteem influenced motivation for doing physical exercise based on social affiliation and social recognition, these had no significant effect on uncontrolled eating. These results confirm that social affiliation promotes positive feelings and healthy life styles [24]. Though previous studies have interpreted social recognition as an extrinsic goal, this study only found a significant effect of dissatisfaction with body image on developing eating disorders and unhealthy eating styles [16,44].

The results of this study have important practical implications. On one hand, self-esteem should be emphasized as a fundamental personal construct for the health and general wellbeing of nursing professionals [12,19]. On the other, the relevance of healthy living habits, such as physical exercise directed at intrinsic goals and good eating behavior, should be underlined because of their effects on the physical and emotional health of workers and the work environment [4,5]. Therefore, organizations, following the recommendations of the HERO model, should design programs designed to promote the health of their employees which are focused on making workers aware of the importance of physical exercise and a balanced diet [21,40]. They should also give workshops on psychological strategies for effective emotional regulation [41,43].

Nevertheless, this study does have some limitations. First, as a cross-sectional study, causal relationships between the variables studied cannot be established, so a longitudinal design would be of interest in future studies. Second, in this study, no gender differences were sought in the reasons for nurses doing physical exercise, and so this question would have to be studied further to design more specialized preventive programs. On the other hand, the data may be skewed due to the collection method, which could lead to certain sources of error, such as simulation [55] and/or negation [56], and there is no doubt that it is related to the honesty of the responses of the workers in the sample. Perhaps it would be advisable to include other qualitative methodologies, such as semi-structured interviews, in the future. Finally, we refer to the need to jointly address the three scales of the TFEQ, through more complex models where there is room, as well as other variables, for emotional eating and cognitive restriction.

Future lines of research should include variables related to the frequency of exercising in addition to variables related to the organization (e.g., engagement). Similarly, multilevel prevention studies would be of interest to analyze whether the level of self-esteem is determined by a healthy life style in different types of work (e.g., employees, supervisors, managers). 

## 5. Conclusions

The main objective of this study was to analyze the mediating role of the reasons for doing exercise in the effect self-esteem has on uncontrolled eating by nursing professionals. This study highlights the importance of self-esteem in complying with healthy life styles that promote physical and emotional wellbeing and the importance of having healthy employees in organizations [5,22]. It is noteworthy that, in the study sample, no data are presented about the clinical severity of uncontrolled eating or the effect that this could have on the health, well-being, or functionality of the professionals. Organizations must understand health in the workplace as well as outside of it, since the better the physical and psychological condition of nursing professionals, the better the healthcare attention of their patients and their families will be, which will thus prevent absenteeism due to health [1].

The most important finding of this study is that the level of self-esteem maintains a relationship with the reasons for doing physical exercise—specifically those related to image, social recognition, and social affiliation. However, in the mediation model, only one significant effect of self-esteem on uncontrolled eating was found, and this was partially mediated by body image. The second most important finding is that low levels of self-esteem directly affect uncontrolled eating in nursing professionals. 

In summary, the results have important practical implications in the framework of Positive Occupational Health Psychology (POHP) by emphasizing self-esteem physical exercise and eating style as essential aspects for the health and wellbeing of workers in the field of healthcare. That is why, based on the results obtained, it is necessary to implement intervention programs from a flexible and dynamic approach. From the network of relationships between the variables, it is possible to propose different lines of action in which, for example, self-esteem and the practice of physical exercise are worked in parallel. In addition, taking into account the benefits of exercise on stress and, in turn, on eating habits, it is possible to design effective interventions that are adaptable to the characteristics of the study population. In this sense, different proposals arise for future lines of research that involve the inclusion of new variables in the analysis (as mentioned above), as well as the examination of the different positions that self-esteem can adopt within the proposed model.

## Figures and Tables

**Table 1:** Descriptive and correlation statistics of self-esteem, physical exercise and uncontrolled eating variables (*N* = 1384).

	*M*	*SD*	1	2	3	4	5	6
1. Self-esteem	32.43	4.52	-					
2. Social affiliation	13.92	5.64	-.01	-				
3. Image	17.83	4.90	-.06 *	.46 ***	-			
4. Health management	21.74	4.45	.16 ***	.38 ***	.51 ***	-		
5. Social recognition	10.87	5.38	-.13 ***	.63 ***	.56 ***	.15 ***	-	
6. Skill development	17.88	5.30	.09 ***	.58 ***	.49 ***	.68 ***	.39 ***	-
7. Uncontrolled eating	17.37	5.80	-.20 ***	.05 *	.18 ***	-.00	.13 ***	.03

* *p* < .05; *** *p* < .001

**Table 2:** Direct, total, and indirect effects.

Self-Esteem and Uncontrolled Eating	*B*	SE	*t*	95% CI
Direct Effect Self-esteem → TFEQ-UE	-.251 ^***^	.038	-6.598	(-.326, -.177)
Total Effect Self-esteem → TFEQ-UE	-.271 ^***^	.038	-7.119	(-.346, -.196)
Ind 1: Self-esteem → IM → TFEQ-UE	-.015	.007		(-.032, -.004)
Ind 2: Self-esteem → IM → S-RE → TFEQ-UE	-.001	.002		(-.009, .002)
Ind 3: Self-esteem → IM → S-AF → TFEQ-UE	.000	.000		(-.000, .003)
Ind 4: Self-esteem → IM → S-RE → S-AF → TFEQ-UE	.001	.001		(-.000, .005)
Ind 5: Self-esteem → S-RE → TFEQ-UE	-.003	.005		(-.015, .005)
Ind 6: Self-esteem → S-RE → S-AF → TFEQ-UE	.002	.002		(-.001, .009)
Ind 7: Self-esteem → S-AF → TFEQ-UE	-.003	.003		(-.014, .001)

Note: Ind: indirect effect, SE = Standard Error, CI = Confidence interval, B = non-standardized regression coefficient; *** *p* < .001. TFEQ-UE (Y) = Uncontrolled Eating; IM (M_1_) = Image; S-RE (M_2_) = Social Recognition; S-AF (M_3_) = Social Affiliation.

## References

[1] Hernández-Vargas C.I., Llorens-Gumbau S., Rodríguez-Sánchez A.M. (2014). Empleados saludables y calidad de servicio en el sector sanitario. Anal. Psicol..

[2] Salanova M., Llorens S., Cifre E., Martínez I.M. (2012). We need a hero! Toward a validation of the healthy and resilient organization (HERO) model. Group Organ. Manag..

[3] Salanova M., Martínez I.M., Llorens S. (2014). A more “positive” look at occupational health from positive organizational psychology during crisis times: Contributions from the WoNT research team. Pap. Psicol..

[4] Salanova M., Llorens S., Martínez I.M. (2016). Contributions from positive organizational psychology to develop healthy and resilient organizations. Pap. Psicol..

[5] Acosta H., Cruz-Ortiz V., Salanova M., Llorens S. (2015). Healthy organization: Analysing its meaning based on the HERO Model/Organizaciones saludables: Analizando su significado desde el Modelo HERO. Rev. Psicol. Soc..

[6] Zurita-Ortega F., Salvador-Pérez F., Knox E., Gámiz-Sánchez V.M.G., Chacón-Cuberos R., Rodríguez-Fernández S., Muros J.J. (2018). Physical activity and health-related quality of life in schoolchildren: Structural equations analysis. Anal. Psicol..

[7] López-Sánchez G.F., Díaz A., Smith L. (2018). Analysis of body image and obesity by Stunkard’s silhouettes in 3-to 18-year-old Spanish children and adolescents. Anal. Psicol..

[8] Moral-García J.E., Orgaz D., López S., Amatria M., Maneiro R. (2018). Influence of physical activity on self-esteem and risk of dependence in active and sedentary elderly people. Anal. Psicol..

[9] Pérez-Fuentes M.C., Molero M.M., Barragán A.B., Martos A., Simón M.M., Gázquez J.J. (2018). Self-efficacy and engagement in health science students and their relation to self-esteem. Int. J. Environ. Res. Public Health..

[10] Bakker A.B., Demerouti E. (2017). Job demands-resources theory: Taking stock and looking forward. J. Occup. Health Psychol..

[11] Lisbona A., Palaci F., Salanova M., Frese M. (2018). The effects of work engagement and self-efficacy on personal initiative and performance. Psicothema.

[12] Molero M.M., Pérez-Fuentes M.C., Gázquez J.J., Barragán A.B. (2018). Burnout in Health Professionals According to Their Self-Esteem, Social Support and Empathy Profile. Front. Psychol..

[13] Orth U., Robins R.W., Meier L.L., Conger R.D. (2016). Refining the vulnerability model of low self-esteem and depression: Disentangling the effects of genuine self-esteem and narcissism. J. Pers. Soc. Psychol..

[14] Bajaj B., Gupta R., Pande N. (2016). Self-esteem mediates the relationship between mindfulness and well-being. Pers. Individ. Differ..

[15] Fulginiti A., Brekke J.S. (2015). Escape from discrepancy: Self-esteem and quality of life as predictors of current suicidal ideation among individuals with schizophrenia. Community Ment. Health J..

[16] Cruz-Sáez S., Pascual A., Wlodarczyk A., Echeburúa E. (2018). The effect of body dissatisfaction on disordered eating: The mediating role of self-esteem and negative affect in male and female adolescents. J. Health Psychol..

[17] Pérez-Fuentes M.C., Gázquez J.J. (2010). Variables relacionadas con la conducta violenta en la escuela según los estudiantes. Int. J. Psychol. Psychol. Ther..

[18] Orth U., Specht J. (2017). The lifespan development of self-esteem. Personality Development across the Lifespan.

[19] Simón M.M., Molero M.M., Pérez-Fuentes M.C., Gázquez J.J., Barragán A.B., Martos Á. (2017). Análisis de la relación existente entre el apoyo social percibido, la autoestima global y la autoeficacia general. Eur. J. Health Res..

[20] World Health Organization (2016). World Health Statistics 2016: Monitoring Health for the SDGs Sustainable Development Goals.

[21] Ross A., Bevans M., Brooks A.T., Gibbons S., Wallen G.R. (2017). Nurses and Health-Promoting Behaviors: Knowledge May Not Translate into Self-Care. AORN J..

[22] Chappel S.E., Verswijveren S.J., Aisbett B., Considine J., Ridgers N.D. (2017). Nurses’ occupational physical activity levels: A systematic review. Int. J. Nurs. Stud..

[23] Annesi J.J., Tennant G.A. (2014). Generalization of theory-based predictions for improved nutrition to adults with morbid obesity: Implications of initiating exercise. Int. J. Clin. Health Psychol..

[24] Saavedra J.M., García-Hermoso A., Escalante Y., Domínguez A.M. (2014). Self-determined motivation, physical exercise and diet in obese children: A three-year follow-up study. Int. J. Clin. Health Psychol..

[25] World Health Organization (2010). Global Recommendations on Physical Activity for Health.

[26] Teixeira P.J., Carraça E.V., Markland D., Silva M.N., Ryan R.M. (2012). Exercise, physical activity, and self-determination theory: A systematic review. Int. J. Behav. Nutr. Phys. Act..

[27] Deci E.L., Ryan R.M. (2000). The “what” and “why” of goal pursuits: Human needs and the self-determination of behavior. Psychol. Inq..

[28] Sebire S.J., Standage M., Vansteenkiste M. (2008). Development and validation of the goal content for exercise questionnaire. J. Sport Exerc. Psychol..

[29] Sebire S.J., Standage M., Vansteenkiste M. (2009). Examining intrinsic versus extrinsic exercise goals: Cognitive, affective, and behavioral outcomes. J. Sport Exerc. Psychol..

[30] Moreno-Murcia J.A., Marcos-Pardo P.J., Huéscar E. (2016). Motivos de práctica físico-deportiva en mujeres: Diferencias entre practiantes y no practicantes. Rev. Psicol. Deporte.

[31] Martos A., Pérez-Fuentes M.C., Molero M.M., Barragán A.B., Simón M.M., Hernández-Garre C.M., Caparrós E.M., Gázquez J.J., Nuñez J.C., Pérez-Fuentes M.C., Molero M.M., Gázquez J.J., Barragán A.B., Simón M.M., Martos A., Hernández-Garre C.M. (2017). Relación entre autoestima y motivación para la práctica deportiva en estudiantes de ciencias de la salud. Perspectivas Psicológica y Educativa de las Necesidades Educativas Especiales.

[32] Pérez-Fuentes M.C., Molero M.M., Simón M.M., Barragán A.B., Martos A., Gázquez J.J. (2018). Emotional intelligence and empathy as predictors of self-efficacy in Certified Nursing Assistants. Rev. Iberoam. Psicol. Salud.

[33] McAuley E., Elavsky S., Motl R.W., Konopack J.F., Hu L., Márquez D.X. (2005). Actividad física, autoeficacia y autoestima: Relaciones longitudinales en adultos mayors. J. Gerontol. B Psychol. Sci. Soc. Sci..

[34] Zamani S.H., Fathirezaie Z., Brand S., Pühse U., Holsboer-Trachsler E., Gerber M., Talepasand S. (2016). Physical activity and self-esteem: Testing direct and indirect relationships associated with psychological and physical mechanisms. Neuropsychiatr. Dis. Treat..

[35] Joseph R.P., Royse K.E., Benitez T.J., Pekmezi D.W. (2014). Physical activity and quality of life among university students: Exploring self-efficacy, self-esteem, and affect as potential mediators. Qual. Life Res..

[36] Booth C., Spronk D., Grol M., Fox E. (2018). Uncontrolled eating in adolescents: The role of impulsivity and automatic approach bias for food. Appetite.

[37] López-Morales J.L. (2018). Eating behaviour analysis and its psychological factors in non-obese university population. Anal. Psicol..

[38] Vainik U., Neseliler S., Konstabel K., Fellows L.K., Dagher A. (2015). Eating traits questionnaires as a continuum of a single concept. Uncontrolled eating. Appetite.

[39] Escandón-Nagel N., Peró M., Grau A., Soriano J., Feixas G. (2018). Emotional eating and cognitive conflicts as predictors of binge eating disorder in patients with obesity. Int. J. Clin. Health Psychol..

[40] Li J., Pursey K., Duncan M., Burrows T. (2018). Addictive Eating and Its Relation to Physical Activity and Sleep Behavior. Nutrents.

[41] Lucena-Santos P., Carvalho S.A., da Silva Oliveira M., Pinto-Gouveia J. (2017). Body-Image Acceptance and Action Questionnaire: Its deleterious influence on binge eating and psychometric validation. Int. J. Clin. Health Psychol..

[42] Dingemans A., Danner U., Parks M. (2017). Emotion regulation in binge eating disorder: A review. Nutrients.

[43] Brechan I., Lundin I. (2015). Relationship between body dissatisfaction and disordered eating: Mediating role of self-esteem and depression. Eat. Behav..

[44] León M.P., González-Martí I., Fernández-Bustos J.G., Contreras O. (2018). Perception of body size and dissatisfaction in children aged 3 to 6: A systematic review. Anal. Psicol..

[45] Kamody R.C., Thurston I.B., Decker K.M., Kaufman C.C., Sonneville K.R., Richmond T.K. (2018). Relating shape/weight based self-esteem, depression, and anxiety with weight and perceived physical health among young adults. Body Image.

[46] Leow S., Jackson B., Alderson J.A., Guelfi K.J., Dimmock J.A. (2018). A Role for Exercise in Attenuating Unhealthy Food Consumption in Response to Stress. Nutrients.

[47] Rosenberg M. (1965). Society and the Adolescent Self-Image.

[48] Ceballos-Ospino G.A., Paba-Barbosa C., Suescún J., Oviedo H.C., Herazo E., Campo-Arias A. (2017). Validez y dimensionalidad de la escala de autoestima de Rosenberg en estudiantes universitarios. Pensam. Psicol..

[49] Stunkard A.J., Messick S. (1985). The three-factor eating questionnaire to measure dietary restraint, disinhibition and hunger. J. Psychosomat. Res..

[50] Jáuregui-Lobera I., García-Cruz P., Carbonero-Carreño R., Magallares A., Ruiz-Prieto I. (2014). Psychometric properties of Spanish version of the Three-Factor Eating Questionnaire-R18 (TFEQ-SP) and its relationship with some eating and body image-related variables. Nutrients.

[51] Pérez-Fuentes M.C., Molero M.M., Gázquez J.J., Oropesa N.F. (2019). Propiedades psicométricas del Three Factor Eating Questionnaire en personal sanitario. Nutr. Hosp..

[52] Preacher K.J., Hayes A.F. (2004). SPSS and SAS Procedures for estimating indirect effects in simple mediation models. Behav. Res. Methods Instrum. Comput..

[53] Preacher K.J., Hayes A.F. (2008). Asymptotic and resampling strategies for assessing and comparing indirect effects in multiple mediator models. Behav. Res. Methods Instrum. Comput..

[54] Baron R.M., Kenny D.A. (1986). The moderator-mediator variable distinction in social psychological research: Conceptual, strategic, and statistical considerations. J. Pers. Soc. Psychol..

[55] Arce R., Fariña F., Vilariño M. (2015). Daño psicológico en casos de víctimas de violencia de género: Un estudio comparativo de las evaluaciones forenses [Psychological injury in intimate partner violence cases: A contrastive analysis of forensic measures]. Rev. Iberoam. Psicol. Salud.

[56] Fariña F., Redondo L., Seijo D., Novo M., Arce R. (2017). A meta-analytic review of the MMPI validity scales and indexes to detect defensiveness in custody evaluations. Int. J. Clin. Health Psychol..

